# Spatial patterns of leaf δ^13^C and δ^15^N of aquatic macrophytes in the arid zone of northwestern China

**DOI:** 10.1002/ece3.7257

**Published:** 2021-02-16

**Authors:** Xusheng Gong, Zhiyan Xu, Qiutong Peng, Yuqing Tian, Yang Hu, Zhongqiang Li, Tao Hao

**Affiliations:** ^1^ Key Laboratory of Development and Environmental Response Faculty of Resource and Environment Hubei University Wuhan China; ^2^ School of Nuclear Technology and Chemistry & Biology Hubei Engineering Research Center for Fragrant Plants Hubei University of Science and Technology Xianning China; ^3^ Taihu Laboratory for Lake Ecosystem Research State Key Laboratory of Lake Science and Environment Nanjing Institute of Geography and Limnology Chinese Academy of Sciences Nanjing China; ^4^ Wildlife Conservation Chief Station of Hubei Province Wuhan China

**Keywords:** aquatic macrophytes, arid zone, climate and environmental factors, patterns, stable isotope composition

## Abstract

Analysis of stable isotope composition is an important tool in research on plant physiological ecology. However, large‐scale patterns of leaf‐stable isotopes for aquatic macrophytes have received considerably less attention. In this study, we examined the spatial pattern of stable isotopes of carbon (δ^13^C) and nitrogen (δ^15^N) of macrophytes leaves collected across the arid zone of northwestern China (approximately 2.4 × 10^6^ km^2^) and attempted to illustrate its relationship with environmental factors (i.e., temperature, precipitation, potential evapotranspiration, sediment total carbon and nitrogen). Our results showed that the mean values of the leaf δ^13^C and δ^15^N in the macrophytes sampled from the arid zone were −24.49‰ and 6.82‰, respectively, which were far less depleted than those measured of terrestrial plants. The order of averaged leaf δ^13^C from different life forms was as follows: submerged > floating‐leaved > emergent. Additionally, our studies indicated that the values of foliar δ^13^C values of all the aquatic macrophytes were only negatively associated with precipitation, but the foliar δ^15^N values were mainly associated with temperature, precipitation, and potential evapotranspiration. Therefore, we speculated that water‐relation factors are the leaf δ^13^C determinant of macrophytes in the arid zone of northwestern China, and the main factors affecting leaf δ^15^N values are the complex combination of water and energy factors.

## Graphical abstract

We investigated the relationships between climate and environmental factors and plant leaf δ^13^C and δ^15^N in the arid zone of northwest China. We speculated that water‐relation factors are the leaf δ^13^C determinant of macrophytes in the arid zone of northwestern China, and the main factors affecting leaf δ^15^N values are the complex combination of water and energy factors.

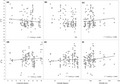

## 1 INTRODUCTION

Stable isotopes in plants, which can indicate how plants have interacted with and responded to their abiotic and biotic environments, have been widely applied in geographical and ecological studies in recent years (Wang et al., 2013). Studies on stable isotopes in plants can not only better determine the relationship between different stable isotope patterns and environmental variables (Cernusak et al., 2013; Li et al., 2017; Yu et al., 2015), but also help to reveal long‐term biogeochemical processes (Evans & Von Caemmerer, 2013; Koba et al., 2003; Leng, 2004).

In recent decades, many studies have demonstrated that leaf‐stable isotope signatures significantly correlate with altitude, latitude, and longitude (Dong et al., 2019; Li et al., 2015, 2017; Zheng & Shangguan, 2007). Some studies showed that leaf carbon isotope ratio (δ^13^C) values of conifers significantly increased with the increasing altitude in the north‐central Rockies (Hultine & Marshall, 2000); whereas, other studies suggested that leaf δ^13^C values of C_3_ plant species in the Loess Plateau of China decreased with increasing altitude (Zheng & Shangguan, 2007). Generally, the foliage nitrogen isotope ratio (δ^15^N) decreases with an increase of altitude. For example, Sah and Brumme (2003) found that the value of leaf δ^15^N of alpine forest in the Kathmandu Valley in Nepal was more depleted at high elevations than at low elevations. However, other individual studies have registered different results. For example, some researchers found that the leaf δ^15^N was not related to altitude in Hawaii Volcano International Park (Vitousek et al., 1989). These inconsistencies suggest that many crucial research questions on leaf‐stable isotope patterns and determinants remain unanswered. Besides, most studies on plant leaf δ^13^C and δ^15^N values have focused on terrestrial ecosystems, and considerably less attention has been devoted to freshwater systems. To the best of our knowledge, few studies have attempted to document large‐scale patterns of foliar‐stable isotope composition, despite aquatic macrophyte being long‐recognized as suitable models for the study of physiological variation (Greulich et al., 2001; Lynn & Waldren, 2001) due to their wide distribution and limited genetic variation (Santamaría et al., 2003). Recently, a few studies have examined regional geographical patterns of leaf δ^13^C values of freshwater macrophytes and indicated that the δ^13^C values displayed a linear increase with the altitude (Li et al., 2015). However, these studies were conducted on a single species and did not consider all macrophytes.

The values of leaf δ^13^C and δ^15^N are sensitive to environmental factors, such as precipitation, temperature, humidity, nutrient, atmospheric pressure, and atmospheric carbon dioxide (CO_2_) concentration (Li et al., 2017; Liu & Wang, 2008). Some studies have shown that leaf δ^13^C is strongly negatively correlated with the mean annual precipitation (Li et al., 2017; Wang et al., 2013). However, another study showed contrary results indicating that leaf δ^13^C of main woody plants in the Gongga Mountain is significantly positively correlated with precipitation (Xie et al., 2014). The effects of temperature on leaf δ^13^C also differ where some effects are positive (Kohls et al., 1994; Loader et al., 1995) while others are negative. This is because low temperatures may not only weaken the photosynthetic enzymatic reactions, which may result in increased ratio of intercellular (ci) to atmospheric (ca) CO_2_ (ci/ca) and decreased leaf δ^13^C values (Arens et al., 2000; Körner et al., 1991), but also reduce the stomal conductance and intercellular CO_2_ concentration, resulting in less depleted δ^13^C values (Körner & Diemer, 1987; Liu & Wang, 2008; Panek & Waring, 1997). For leaf δ^15^N, several studies have proved that the correlation of leaf δ^15^N with annual precipitation differ across different types of ecosystems. Very positive δ^15^N values have often been reported in arid areas (Lajtha & Schlesinger, 1986; Schulze et al., 1991), while negative values have been reported in high rainfall or cold and wet systems outside the mainland tropics (Nadelhoffer et al., 1996). Previous studies have reported conflicting conclusions on the potential changes in the mean annual temperature to influence δ^15^N. Most studies have suggested that high temperatures could strengthen soil‐nitrifying bacteria and ammonifying bacterial activities, resulting in soil N pool enriched in ^15^N and increased leaf δ^15^N values (Amundson et al., 2003; Martinelli et al., 1999). However, some researchers have also insisted that the relationship between the leaf δ^15^N and the annual average temperature is not a simple linear relationship on a global scale (Craine et al., 2009). However, most studies on the pattern of plant leaf δ^13^C and δ^15^N have focused on terrestrial ecosystems and considerably less attention has been devoted to freshwater systems.

In addition, previous studies have indicated that plant life forms of aquatic macrophytes can significantly influence the variation in leaf‐stable isotope composition due to their different available photosynthetic carbon sources (Liu et al., 2020; Yu et al., 2015). Most of the researches on leaf δ^13^C of aquatic plants focuses on the inorganic carbon in water (Li et al., 2011), the pH value of water (Liu et al., 2020; Maberly, 1996), seasonal changes (Cloern et al., 2002), etc. Although the leaf δ^15^N of aquatic macrophytes is mainly used as an indicator of dissolved inorganic nitrogen (DIN) in the water ecosystem (Cole et al., 2004; Wen et al., 2010), its relationship with sediment is less explored. Moreover, most of these studies are small–medium scale studies covering a single lake or river. Therefore, there is an urgent need to study leaf patterns in freshwater assemblages at different scales to discover whether they follow the general patterns found in terrestrial ecosystems.

In the arid zone of northwestern China, extreme aridity gradients exist over relatively short geographical distances in both the east–west and north–south regions (Feng et al., 1989; Tang et al., 1992). In such environmental transects, plants encounter various microclimates differing in temperature, soil moisture, and vapor pressure gradients, each of which may influence the variation in the leaf δ^13^C and δ^15^N. Moreover, there are clear environmental factors such as gradients of water quantity and water availability, and macrophytes can adapt well to complex aquatic environments. Thus, we hypothesize that leaf δ^13^C and δ^15^N values of aquatic macrophytes in the arid zone of northwest China are largely affected by plant‐life forms and shift consistently along environmental gradients. The main objectives of the present study are as follows: (a) analyze the differences in the leaf δ^13^C and δ^15^N of different life forms; (b) describe the patterns of leaf δ^13^C and δ^15^N of the aquatic macrophytes along a large geographical gradient; and (c) assess the relationships between variation in leaf δ^13^C and δ^15^N values and environmental, climatic factors (i.e., temperature, precipitation, potential evapotranspiration, sediment total carbon and nitrogen). We strongly believe that this study can help to elucidate the physiological effects of natural selection and reveals different strategies of aquatic macrophytes adaptive potential.

## 2 MATERRIAL AND METHODS

### 2.1 Study area

**FIGURE 1:**
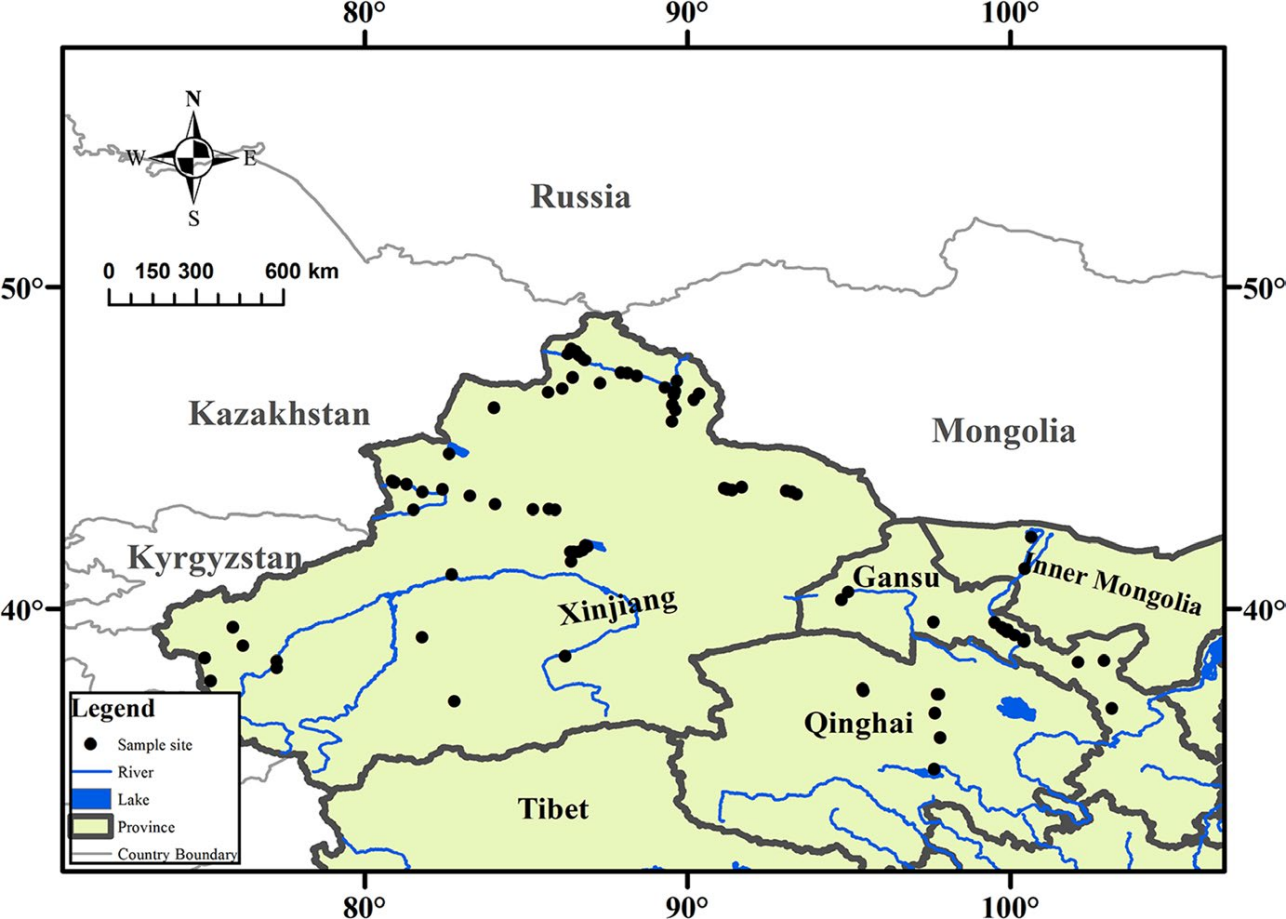
Map showing the sites at which the aquatic plants were collected and their location in the arid zone of northwestern China

The arid zone (35°–49°N, 73°–106°E) is a land‐locked region located in northwestern China (Figure 1) and is surrounded by the Qinghai–Tibet Plateau and many high mountains. The climate is generally water‐limited, and steppe biomes are prevalent. The annual rainfall in the arid zone is <250 mm, with certain areas receiving <100 mm annually, but the annual evaporative capability is above 2,000 mm. The mean annual temperature is 2–6°C, with a maximum monthly mean temperature above 28°C, a minimum monthly mean temperature below −16°C, and a daily temperature that fluctuates significantly (up to 20°C) (Feng et al., 1989).

### 2.2 Sampling and measurements

The aquatic macrophyte collections were conducted in the arid zone pristine area (no human pressure on water bodies) from July to October 2011. The sampling sites in this study covered almost the entire arid zone of northwestern China, and the altitude varied from 313 to 3,535 m above sea level (Figure 1).

In this study, 131 aquatic plant samples from 45 kinds of aquatic plants and 79 sampling sites across the arid area of northwestern China were collected (Figure 1, Table 1). This included 71 emergent plants, 20 floating‐leaved plants, and 40 submerged plants. There are three replicates per sample. The plants collected at each site were placed in paper envelopes and dried in the sun. At each sampling site, the latitude, longitude, and altitude were recorded using a global positioning system (GPS). Surface sediments where macrophytes grew were collected from the top 0–10 cm layers of undisturbed sediments with a columnar sampling instrument. At each sites, 3 sediments were collected and mixed together, then put into paper envelopes and dried in the sun. The samples of macrophyte and sediment were dried to a constant mass at 60°C for 72 hr in an oven upon returning to the laboratory. All the dried samples of macrophytes and sediments were ground to a fine powder with a mortar in the laboratory. The total C and N contents of macrophyte and sediment were determined with an elemental analyzer (NA2500, Carlo Erba Reagenti). The total P of macrophytes was measured using a sulfuric acid/hydrogen peroxide digest and the ammonium molybdate ascorbic acid method (Richard & Donald, 1996). The stable isotope ratios were expressed in denotation as parts per thousand (m) deviation from the international standards according to the following equations:

$$\delta^{13}C=\left[{{(}^{13}C{/}^{12}C)}_{\text{sample}}/{{(}^{13}C{/}^{12}C)}_{{\text{standard}}^{‐1}}\right]\times 1,000$$

$$\delta^{15}N=\left[{{(}^{15}N{/}^{14}N)}_{\text{sample}}/{{(}^{15}N{/}^{14}N)}_{{\text{standard}}^{‐1}}\right]\times 1,000$$

where ^15^N/^14^N are the isotopic ratios of the sample and standard (atmospheric nitrogen), and ^13^C/^12^C are the isotopic ratios of the sample and PDB (Pee dee Belemnite formation from South Carolina, USA) standard. The average standard deviation of replicate measurements for δ^13^C and δ^15^N was 0.3‰.

**TABLE 1:** List of aquatic macrophytes and their sampling times in the arid zone of northwestern China

Species	Life form	Sampling times	Species	Life form	Sampling times
*Alisma orientale*	Emergent	9	*Potamogeton crispus*	Submerged	2
*Batrachium bungei*	Submerged	4	*Potamogeton distinctus*	Floating‐leaved	3
*Butomus umbellatus*	Emergent	4	*Potamogeton heterophyllus*	Submerged	3
*Callitriche stagnalis*	Submerged	1	*Potamogeton lucens*	Submerged	2
*Ceratophyllum demersum*	Submerged	4	*Potamogeton natans*	Floating‐leaved	3
*Cyperus difformis*	Emergent	1	*Potamogeton obtusifolius*	Submerged	1
*Epilobium hirsutum*	Emergent	2	*Potamogeton oxyphyllus*	Submerged	2
*Halerpestes cymbalaria*	Emergent	1	*Potamogeton pectinatus*	Submerged	4
*Heleocharis dulcis*	Emergent	1	*Potamogeton perfoliatus*	Submerged	6
*Hippuris vulgaris*	Emergent	5	*Ranunculus natans*	Floating‐leaved	7
*Juncus alatus*	Emergent	3	*Ranunculus sceleratus*	Emergent	2
*Lemna minor*	Floating‐leaved	2	*Sagittaria trifolia*	Emergent	1
*Limosella aquatica*	Emergent	2	*Scirpus validus*	Emergent	1
*Myriophyllum verticillatum*	Submerged	2	*Sparganium simplex*	Emergent	2
*Myriophyllum spicatum*	Submerged	3	*Sparganium stoloniferum*	Emergent	8
*Najas graminea*	Submerged	1	*Triglochin maritimum*	Emergent	1
*Najas marina*	Submerged	3	*Triglochin palustre*	Emergent	3
*Nymphaea tetragona*	Floating‐leaved	1	*Typha davidiana*	Emergent	1
*Nymphoides peltatum*	Floating‐leaved	1	*Typha gracilis*	Emergent	1
*Oenanthe javanica*	Emergent	2	*Typha orientalis*	Emergent	11
*Phragmites australis*	Emergent	3	*Veronica undulata*	Emergent	6
*Polygonum amphibium*	Floating‐leaved	3	*Zannichellia palustris*	Submerged	2
*Polygonum hydropiper*	Emergent	1			

### 2.3 Environment variables

The mean annual precipitation (MAP), mean annual temperature (MAT), and potential evapotranspiration (PET) were selected to test the effects of water‐energy factors on foliar stable isotope composition. Precipitation data were obtained from the Climate Hazards Group database (CHIRPS) at a spatial resolution of 0.05° from (http://chg.geog.ucsb.edu). Temperature data were obtained from NOAA Earth System Research Laboratory's Physical Sciences Division (PSD) at a spatial resolution of 0.5° from (https://www.esrl.noaa.gov). PET data were obtained from the MOD16A3 product at a 1‐km spatial resolution (http://files.ntsg.umt.edu/data/NT SG_Products/MOD16/). MAP from 1981 to 2015, MAT from 2010 to 2014, and mean annual PET from 2000 to 2014 of each sampling site were extracted from the abovementioned downloaded data using ArcGIS 10.4.1 software with the “Extract values to points” function. MAP, MAT, and PET data for each sample site were obtained from the mean value of the MAP with a record length of 35 years, MAT with a record length of 5 years, and mean annual PET with a record length of 15 years.

### 2.4 Data analysis

Statistical analyses were performed using the statistical software SPSS Statistics 19 (IBM). The mean and standard deviation (*SD*) of leaf δ^13^C, δ^15^N, C%, N%, and P% were calculated for all species and each life form. Analysis of variance (ANOVA) was applied to determine the statistical significance of the differences in the leaf δ^13^C and δ^15^N of different life forms, which was test with post hoc contrasts using the Student–Newman–Keuls test. Before performing a one‐way ANOVA, all the data were tested for normality and homogeneity. Non‐normal data were transformed (log10) to obtain normality. Linear regression analyses were used to test the relationships between leaf δ^13^C and δ^15^N and C, N, P contents, and N:P ratio overall and in different macrophyte life forms. Univariate linear regression analyses were performed to examine the effects of environmental variables on leaf δ^13^C and δ^15^N. Additionally, general linear models (GLMs) were applied to test the effects of MAT, MAP, PET, sediment total carbon (STC), sediment total nitrogen (STN), and life forms on foliar δ^13^C and δ^15^N.

## 3 RESULTS

### 3.1 The leaf δ^13^C and δ^15^N values and the relationship between leaf δ^13^C and δ^15^N and foliar C, N, and P content

The leaf δ^13^C and δ^15^N of the macrophytes collected from the arid zone of northwestern China varied widely. The foliar δ^13^C values of all the macrophytes ranged from −32.38‰ to −12.55‰, with a mean value of −24.49‰. The mean leaf δ^15^N was 6.82‰, with a range of −3.41‰ to 17.43‰ (Table 2). Among the different life forms, the submerged plants had the most enriched leaf δ^13^C, with a mean value of −20.31‰. Emergent plants had the most depleted leaf δ^13^C, with a mean value of −26.75‰. For foliar δ^15^N, the floating‐leaved macrophytes had the most enriched value, with a mean value of 7.65‰ (Table 2). One‐way ANOVA showed that there were significant differences in foliar leaf δ^13^C of the submerged, floating‐leaved, and emergent plants in the study area (Table 2).

**TABLE 2:** Leaf δ^13^C, δ^15^N, C%, N%, and P% (mean ± *SD*) overall and of the three macrophyte life forms in the arid zone of northwestern China (significant differences between the means within the rows, *p* < .05, are indicated by different letters)

	Overall	Life form		
		Submerged	Floating‐leaved	Emergent
*n*	131	40	20	71
δ^13^C‰	−24.49 ± 4.30	−20.31 ± 5.03c	−24.84 ± 2.48b	−26.75 ± 1.84a
δ^15^N‰	6.82 ± 3.49	6.69 ± 2.97b	7.65 ± 4.77a	6.66 ± 3.36b
C%	40.70 ± 3.30	38.96 ± 3.33b	41.43 ± 2.68a	41.48 ± 3.10a
N%	3.18 ± 0.99	3.23 ± 0.80b	3.63 ± 1.22a	3.02 ± 0.99c
P%	0.17 ± 0.12	0.17 ± 0.12b	0.22 ± 0.19a	0.15 ± 0.09b

Our results also showed that there were significant differences in foliar leaf C, N, and P contents of the collected submerged, floating‐leaved, and emergent plants. Among the three macrophyte life forms, the floating‐leaved plants had less depleted leaf N% and P% than those of the other two aquatic plant life forms (Table 2).

The linear regression analyses indicated that there were only significant negative correlations between the foliar δ^13^C and C% of all the macrophytes (*y = *−0.42*x = *7.25, *r* ^2^ *= *.11, *p* < .001) and foliar δ^13^C and N% of the emergent plants (*y = *−0.62*x = *28.63, *r* ^2^ *= *.11, *p* < .001).

### 3.2 Patterns of leaf δ^13^C and δ^15^N of aquatic macrophytes across northwestern China

**FIGURE 2:**
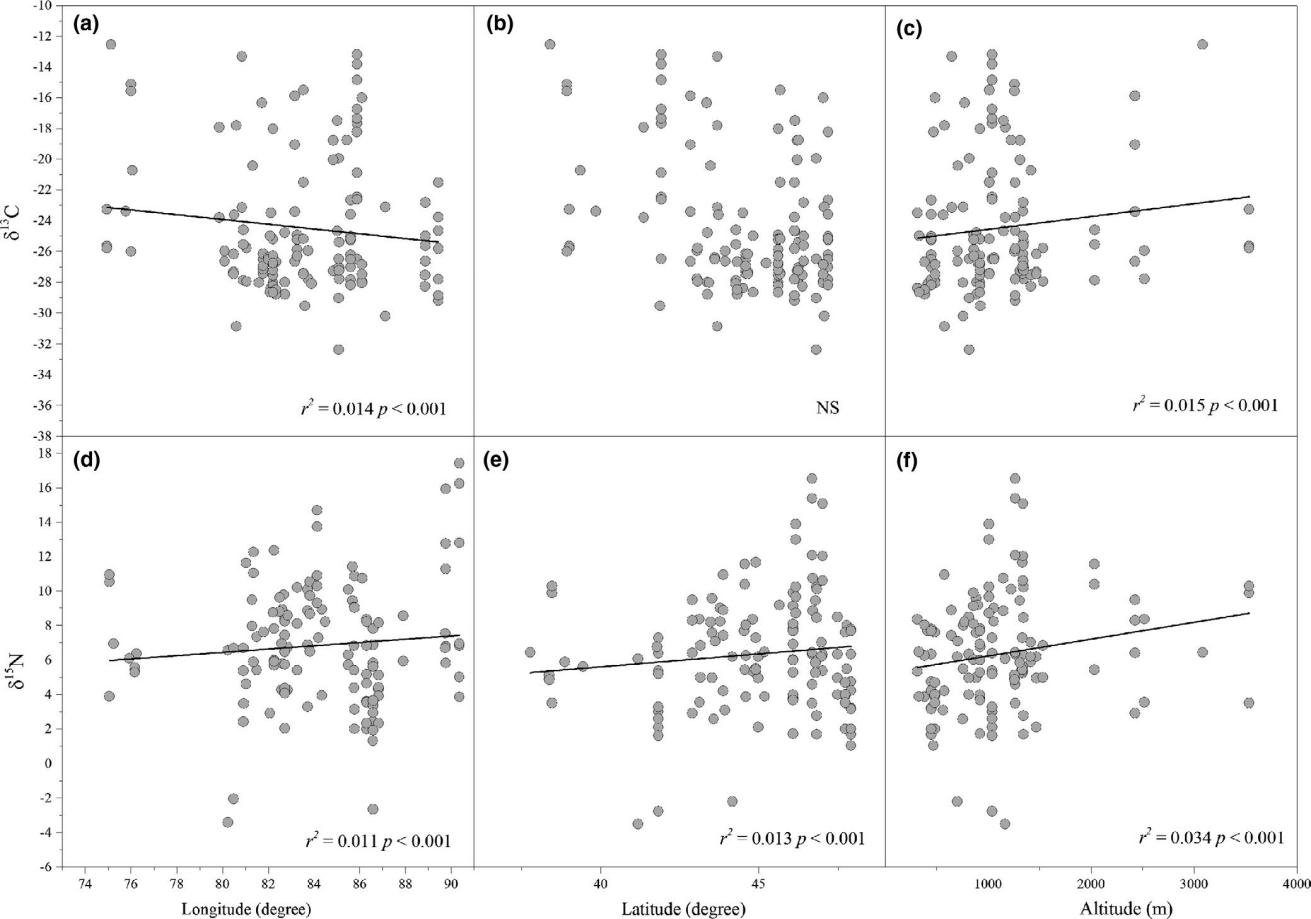
The relationships between leaf δ^13^C and δ^15^N of all the macrophytes and longitude, latitude and altitude (NS, *p* > .05)

The linear regression indicated that the leaf δ^13^C of all the macrophytes was negatively associated with longitude but positively associated with altitude, and the leaf δ^15^N of all the macrophytes exhibited positive correlations with longitude, latitude, and altitude (Figure 2).

The leaf δ^13^C of the submerged plants was negatively associated with latitude but positively associated with altitude. The leaf δ^13^C of the floating leaves was negatively associated with latitude, and the leaf δ^15^N of the floating leaves was positively associated with latitude (Table 3).

**TABLE 3:** shows the relationships between the leaf δ^13^C and δ^15^N of all the species and different macrophyte life forms and longitude, latitude, and altitude

	*Slope*			
	Life forms	Longitude	Latitude	Altitude
δ^13^C	Submerged	ns	−0.79**	0.003*
	Floating‐leaved	ns	−0.41*	ns
	Emergent	ns	ns	ns
δ^15^N	Submerged	ns	ns	ns
	Floating‐leaved	ns	0.73*	ns
	Emergent	ns	ns	ns

Note

ns: *p* > .05.

** *p* < .01.

* *p* < .05.

### 3.3 Relationships between the leaf δ^13^C and δ^15^N and environmental variables

General linear models indicated that life form only significantly affected foliar δ^13^C but not foliar δ^15^N. Among the three climatic variables tested, only MAP significantly influenced the leaf δ^13^C of all the species. However, all the tested variables, including MAT, MAP, and PET, significantly negatively influenced the leaf δ^15^N of all the macrophytes (Table 4).

**TABLE 4:** Summary statistics of the general linear models, which show the effects of multi‐annual mean temperature (MAT, °C), multi‐annual mean precipitation (MAP, mm), potential evapotranspiration (PET, mm), sediment total carbon (STC), sediment total nitrogen (STN), and life forms on leaf δ^13^C and δ^15^N of all the species and the three different macrophyte life forms

	Variables	*df*	δ^13^C	δ^15^N
			B	B
All species	MAT	1	−0.28	−0.77***
	MAP	1	−0.03*	−0.05***
	PET	1	3.03 × 10^–5^	−5.08 × 10^–5^ ***
	STC	1	−0.02	0.03
	STN	1	0.12	0.19
	Life forms	2	−2.44***	−0.12
	MAT	1	−1.24*	−0.86**
	MAP	1	−0.09*	−0.06**
Submerged macrophytes	PET	1	5.35 × 10^–5^	−8.13 × 10^–5^ **
	STC	1	−0.06	−0.01
	STN	1	1.56	1.03
	MAT	1	−0.55***	−0.77
	MAP	1	−0.04***	−0.01
Floating‐leaved macrophytes	PET	1	1.92 × 10^–5^	−4.63 × 10^–5^
	STC	1	−0.02	0.02
	STN	1	0.45	−0.85
	MAT	1	0.18	−0.79***
	MAP	1	0.01	−0.06***
Emergent macrophytes	PET	1	2.35 × 10^–5^	−6.27 × 10^–5^ *
	STC	1	−0.01	0.02
	STN	1	−0.15	0.32

*** *p* < .001.

** *p* < .01.

* *p* < .05.

The leaf δ^13^C values of submerged and floating‐leaved macrophytes were negatively associated with MAT and MAP. Additionally, there was a significantly negative relationship between MAT, MAP, and PET and foliar δ^15^N of the submerged plants and emergent plants (Table 4).

Also, our results showed that no statistically significant correlation relationships were found between the leaf δ^13^C and δ^15^N value and STC and STN (Table 4).

## 4 DISCUSSION

### 4.1 The leaf δ^13^C and δ^15^N values of different groups and relationships between leaf stable isotope signatures and C%, N%, and P% in the arid zone of northwestern China

In the present study, the mean leaf δ^13^C value of aquatic macrophytes in the arid zone of northwestern China was −24.49‰. It was less depleted than the mean leaf δ^13^C values of terrestrial plants reported by Li et al. (2017) from 2,538 observations in China (−27.15‰) and by Kohn (2010) from approximately 570 sites on a global scale (−27.0‰). However, considering the different life forms, the leaf δ^13^C values of emergent macrophytes were almost the same as the δ^13^C values reported by Li et al. (2017) and by Kohn (2010), and the mean leaf δ^13^C of the submerged plants was markedly less depleted than that of the emergent plants and terrestrial plants. This result may be attributable to the unique aerenchyma and C‐uptake mechanism of macrophytes, which is different from that of terrestrial plants (Pedersen et al., 2013). Different photosynthetic carbon sources may be another main reason for the differences in leaf δ^13^C values among the three life forms and terrestrial plants (James & Larkum, 1996). Compared with terrestrial and emergent plants using a single form of inorganic carbon (CO_2_) derived from a large, well‐mixed atmospheric reservoir, submerged plant derives inorganic carbon from dissolved inorganic carbon (DIC) which may be comprised mostly of aqueous CO_2_ at a pH < 6.4, bicarbonate (${\text{HCO}}_{3}^{‐}$) at a pH of between 6.4–10.3, or carbonate (${\text{CO}}_{3}^{2‐}$) at a pH > 10.3 (Finlay & Kendall, 2008). Each carbon sources has a distinct δ^13^C. A previous study found that the δ^13^C value of ${\text{HCO}}_{3}^{‐}$ was 7‰–11‰ higher than that of CO_2_ (Stephenson et al., 1984). Thus, for foliar δ^13^C of different life form, it is reasonable that submerged plants have the most enriched value, followed by floating‐leaved plants, and emergent plants. Additionally, significant differences in the δ^13^C values among the three life forms may reflect significant functional changes in the metabolism of macrophytes. The correlation between the foliar δ^13^C values of the emergent plants and foliar N% suggested that variations in the foliar δ^13^C values of the emergent plants were likely caused by nutrient‐related changes in photosynthetic capacity rather than by stomatal limitation (Ma et al., 2005).

Our results indicated that the mean content of leaf δ^15^N was 6.82‰, which is significantly less depleted than that of herb samples observed in China (Fang et al., 2011; Liu et al., 2018). Previous studies have shown that the nitrogen isotope values of plants are mainly related to the availability of nitrogen and the demand for nitrogen in plants (Dawson et al., 2002; Evans, 2001). The δ^15^N of aquatic plants is controlled by the type of dissolved inorganic nitrogen utilized, its δ^15^N value, and the fractionations associated with discrimination against ^15^N during N uptake that may vary by plant species and environmental conditions (Finlay & Kendall, 2008). The relatively enriched mean leaf δ^15^N values of the aquatic macrophytes may be an adaptation strategy to nitrogen deficiency due to low soil N mineralization and low N leaching in arid ecosystems (Geng et al., 2014). Some studies have indicated that when nitrogen availability is lower than the nitrogen requirement of plants, δ^15^N fractionation is inhibited during absorption assimilation, resulting in less depleted δ^15^N of aquatic plants (Yu et al., 2015).

### 4.2 Relationships between the leaf δ^13^C and δ^15^N and environmental and geographical factors

Environmental factors, such as energy, precipitation, water physicochemical factors, and soil nutrients can significantly affect leaf δ^13^C and δ^15^N characteristics (Anderson et al., 2000; Brooks et al., 1997; Liu & Wang, 2008; Leng, 2004). Energy factors, including MAT and PET, are important environmental factors that influence leaf stable isotopes by directly affecting stomatal control, physiological processes, and CO_2_ fixation (Leavitt & Long, 1983; Martinelli et al., 1999; Warren & Adams, 2000; Yi & Yang, 2006). Our study showed that MAT negatively affected the leaf δ^15^N of all the aquatic macrophytes, submerged plants, and emergent plants, which is inconsistent with research results in most terrestrial plants (Amundson et al., 2003; Martinelli et al., 1999; Yi & Yang, 2006). This could be due to the increased temperature hinders the migration of oxygen to deep water, causing the underwater to gradually enter the environment of hypoxia or even anaerobic, which is beneficial to the metabolic conversion of denitrifying bacteria, resulting in reduced concentration of N in the water (Cornelia & Valentina, 2016; Liu et al., 2016). Similarly, PET displayed strong negative correlations with leaf δ^15^N in all the aquatic macrophytes, submerged plants, and emergent plants, which may be explained by a slower rate of runoff in the arid ecosystem. When the PET increases, the evaporation of water and the decrease of runoff rate reduced the N leaching into water, eventually leading to a decrease in plant δ^15^N. Our results also showed that the leaf δ^13^C of submerged and floating‐leaved plants were negatively related to MAT. A possible explanation is that low temperatures could weaken the photosynthetic enzymatic reactions, resulting in increased ci/ca and decreased leaf δ^13^C values (Arens et al., 2000).

Precipitation is considered to be another crucial factor that influences the leaf‐stable isotope signature (Diefendorf et al., 2010; Kohn, 2010; Li et al., 2017; Swap et al., 2004). Our findings indicated that MAP presented strong negative correlations with the leaf δ^15^N of all the aquatic macrophytes, submerged plants, and emergent plants. Many studies have shown that precipitation affects plant δ^15^N, which is mainly related to the influence of precipitation on the conversion of the soil organic N pool to the inorganic N pool, and inorganic N loss is due to the volatilization process and the denitrification process directly fractionating ^15^N (Craine et al., 2009; Groffman et al., 1993; Swap et al., 2004). Some studies have shown that δ^15^N of aquatic plants can be affected by anthropogenic pollution (Cole et al., 2004; Fry et al., 2000). However, we did not examine the relationships between the leaf δ13C and δ15N and water quality because of the little human pollution and the difficulty to preserve the water sample for long periods during field investigation. Nevertheless, our results suggested that no significant correlations were found between the leaf δ^15^N value and foliar N content and STN. Thus, we speculate the leaf δ^15^N value of macrophytes in the arid zone of northwestern China were water‐limited. This conclusion is consistent with previous findings that the leaf δ^13^C values were extremely limited by precipitation (Li et al., 2015; Ma et al., 2005; Zheng & Shangguan, 2007). Our results showed that leaf δ^13^C values of all the aquatic macrophytes, submerged plants, and floating‐leaved plants were negatively correlated with precipitation. This could be due to the effect of precipitation on relative humidity and moisture availability because there are significant differences in water availability for different aquatic habitat due to low precipitation, high evaporation, high pH, salinity, and conductivity of the water body in the arid zone (Feng et al., 1989).

Longitude, latitude, and altitude do not affect plant‐stable isotope composition per se, but rather influence related environmental factors. In the arid zone of northwestern China, we found that the leaf δ^13^C values of all the aquatic macrophytes were associated with altitude and longitude, which were negatively associated with MAP (Figure 2, Table 4). Therefore, we inferred that altitude and longitude affected the foliar δ^13^C values of the macrophytes mainly via the joint effects of water factors. Additionally, our results showed that latitude, altitude, longitude, MAT, MAP, and PET were associated with the foliar δ^15^N values of all the aquatic macrophytes (Figure 2, Table 4). These results implied that latitude, altitude, and longitude could affect foliar δ^15^N values of macrophytes through a complex combinations of water and energy factors.

## 5 CONCLUSION

In this study, we found that the mean leaf δ^13^C and δ^15^N values were far less depleted in aquatic macrophytes than in terrestrial plants, and submerged plants had the most enriched mean leaf δ^13^C values among the three life forms. Our results also showed strong relationships between leaf δ^13^C and longitude and altitude, as well as between leaf δ^15^N and longitude, latitude, and altitude. Our findings indicated that foliar δ^13^C values were mainly associated with MAP, while foliar δ^15^N values were mainly associated with MAT, PET, and MAP, which was consistent with our prediction that water quantity and water availability factors drive foliar δ^13^C and δ^15^N values of macrophytes in the study area. Complex environmental factors, such as water quality, CO_2_ supply, and daylight hours may also be important factors influencing the spatial distribution of the leaf‐stable isotope composition of aquatic macrophytes. In this study, we only evaluated the effect of geographic factors (longitude, latitude, and altitude), climate factors (MAT, PET, and MAP), and sediment total carbon and nitrogen on leaf δ^13^C and δ^15^N values of aquatic macrophytes. Numerous studies have shown that δ^15^N of aquatic plants are associated with N sources, such as pore‐waters DIN and pollution (Cole et al., 2004; Fry et al., 2000). Thus, further research should investigate the effects of other factors on the leaf‐stable isotope composition of macrophytes.

## CONFLICT OF INTEREST

None declared.

## AUTHOR CONTRIBUTIONS

**Xusheng Gong:** Data curation (equal); Formal analysis (equal); Funding acquisition (supporting); Investigation (equal); Methodology (equal); Software (equal); Visualization (equal); Writing‐original draft (equal); Writing‐review & editing (equal). **Zhiyan Xu:** Investigation (supporting); Validation (supporting); Visualization (supporting). **Qiutong Peng:** Investigation (supporting); Visualization (supporting); Writing‐original draft (supporting). **Yuqing Tian:** Investigation (supporting). **Yang Hu:** Formal analysis (supporting); Funding acquisition (supporting); Investigation (supporting). **Zhongqiang Li:** Conceptualization (equal); Data curation (equal); Funding acquisition (equal); Methodology (equal); Project administration (equal); Resources (equal); Supervision (equal); Writing‐original draft (equal); Writing‐review & editing (equal). **Tao Hao:** Conceptualization (equal); Data curation (supporting); Supervision (supporting); Writing‐review & editing (supporting).

## Data Availability

Data are available from the Dryad Digital Repository: https://doi.org/10.5061/dryad.7m0cfxpst
